# Hydropneumothorax Post COVID-19 Infection

**DOI:** 10.7759/cureus.27827

**Published:** 2022-08-09

**Authors:** Swetha Vennelaganti, Aditya Sanjeevi, Sathyamurthy P, Basith Ahamed NM

**Affiliations:** 1 Internal Medicine, Sri Ramachandra Institute of Higher Education and Research, Chennai, IND; 2 General Medicine, Sri Ramachandra Institute of Higher Education and Research, Chennai, IND

**Keywords:** steroids in covid-19, acute worsening in covid-19, pleuritic pain in covid-19, complications of covid-19, covid-19, hydropneumothorax

## Abstract

A 32-year-old male presented to the hospital with chief complaints of fever, cough, and breathlessness for the past 4 days and was found to be positive for severe acute respiratory syndrome coronavirus 2 (SARS-CoV-2). On arrival at the hospital, the patient required supplemental oxygen. In addition, injection enoxaparin 80 mg subcutaneous twice a day and injection methylprednisolone 40 mg IV twice a day were administered for 10 days. Following this, the patient reported symptomatic improvement and was shifted to the ward with O2 @ 2 L/min through nasal prongs. However, the same evening he complained of right-sided pleuritic chest pain and developed worsening hypoxemia. CT scan of the thorax confirmed the presence of hydropneumothorax with a mediastinal shift to the left side. An intercostal drain (ICD) was placed after shifting him to the intensive care unit (ICU); pleural fluid sent for analysis confirmed the presence of a secondary bacterial infection for which he was treated with appropriate parenteral antibiotics.

## Introduction

The spectrum of coronavirus disease 2019 (COVID-19) infection ranges from asymptomatic infection to respiratory failure requiring mechanical ventilation. There are only a few case reports of COVID-19 patients developing pneumothorax and only one case of hydropneumothorax has been reported to date. Our patient is the second reported case of hydropneumothorax post-COVID-19 infection. Spontaneous pneumothorax may occur in COVID-19 patients due to rupture of a subpleural bleb and is considered to be more common in those receiving positive pressure ventilation. However, our patient did not receive any positive pressure ventilation and was in the fourth week of illness with his symptoms abating. Even in such a patient, the development of sudden onset pleuritic chest pain, dyspnea along with hypoxemia, a diagnosis of pneumothorax should be kept in mind and the development of hydropneumothorax in such patients may point towards a superadded bacterial infection.

## Case presentation

**Figure 1:**
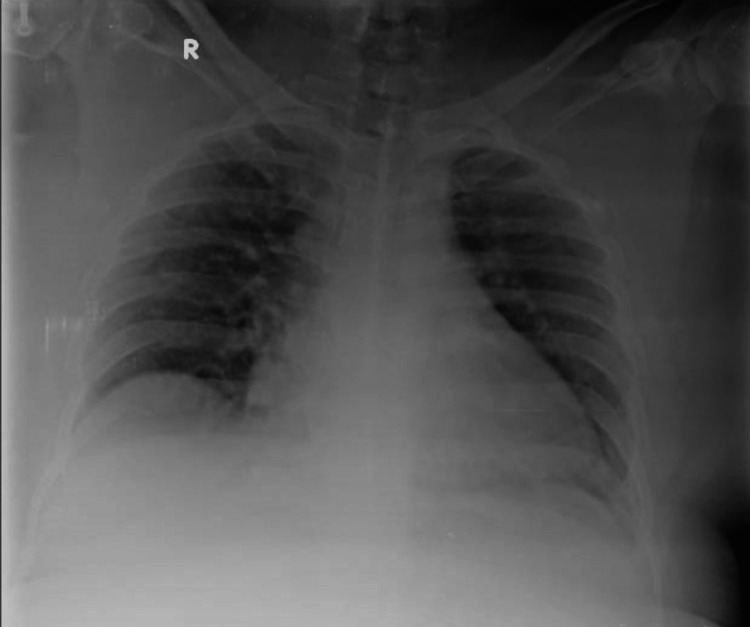
Admission chest X-ray revealing patchy airspace opacities.

**Figure 2:**
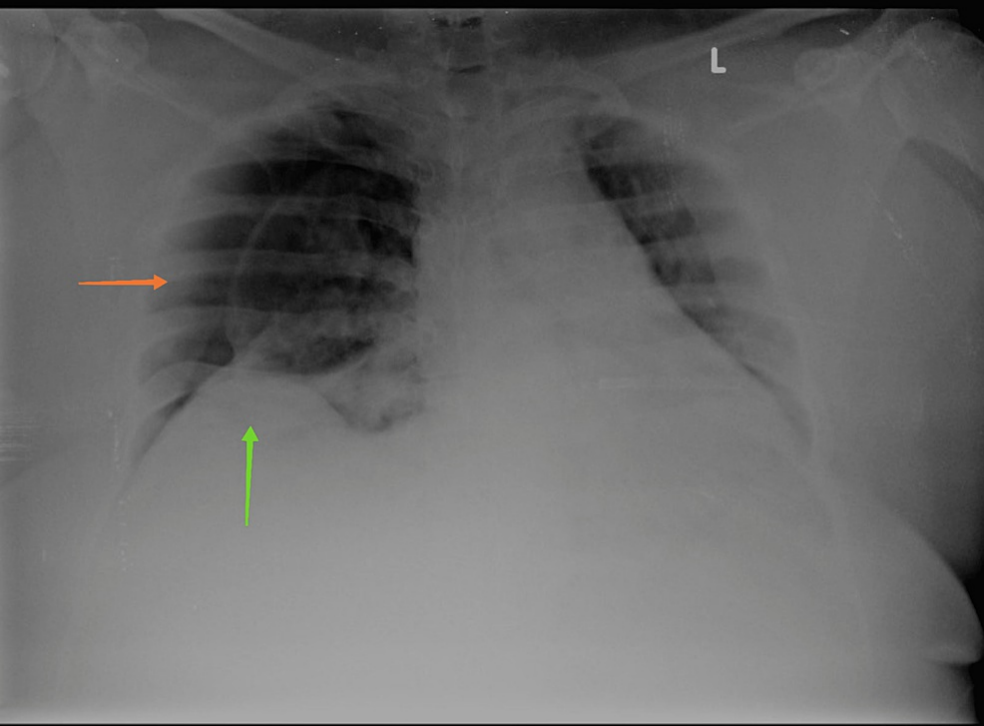
Right-sided pneumothorax with right lower lobe collapse.

**Figure 3:**
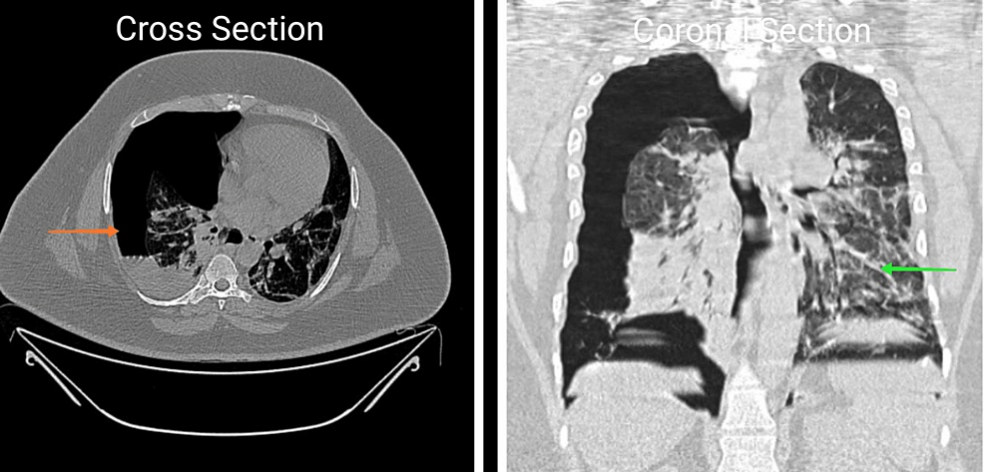
CT thorax.

**Figure 4:**
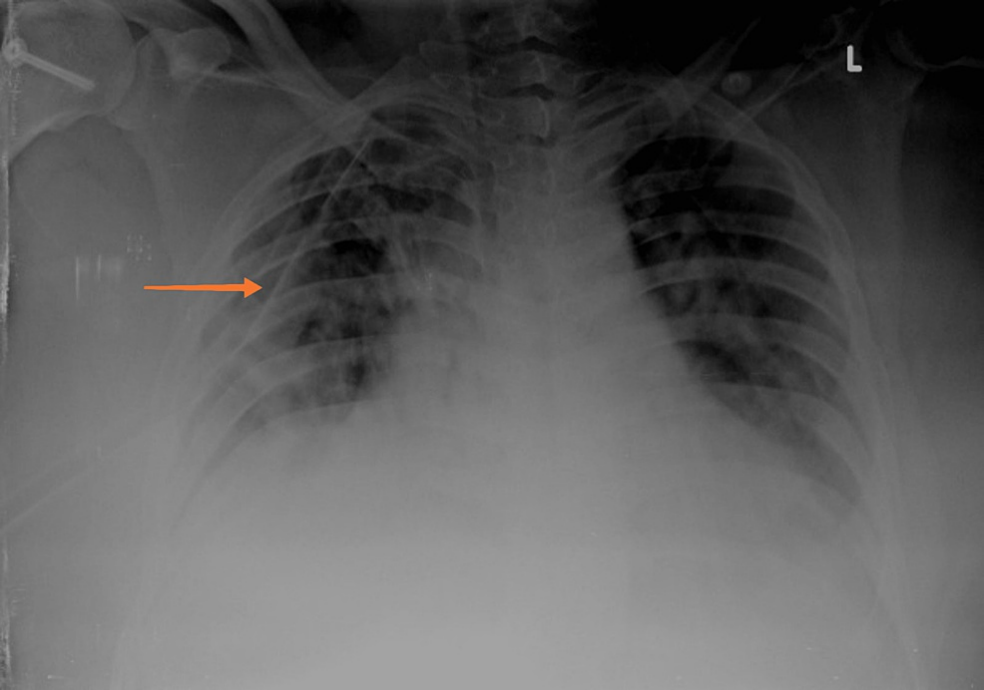
Chest X-ray: pneumothorax with ICD in situ in the right pleural space. ICD, intercostal drain

**Figure 5:**
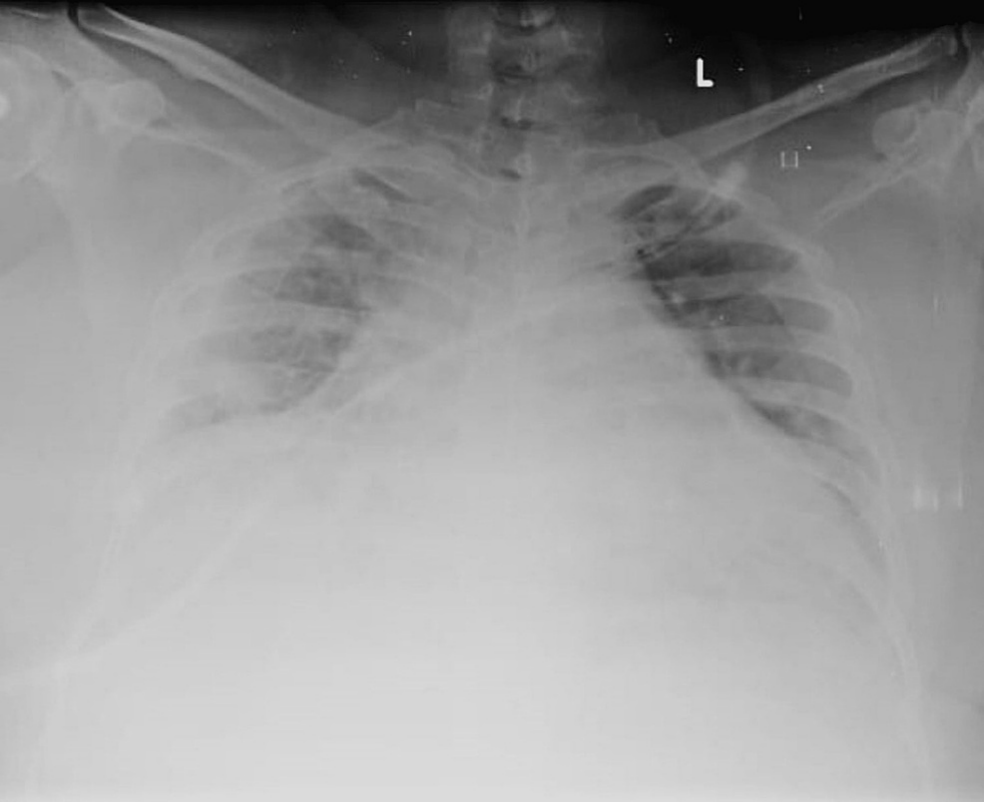
Chest X-ray at discharge: re-expansion of the right lung field.

A 32-year-old male came with complaints of fever, cough with minimal expectoration, and dyspnea on exertion for the past 4 days. A history of contact with a COVID-19-positive patient at his workplace was present. In addition, the patient also had a 10-pack-year smoking history but no previous pulmonary pathology. He was morbidly obese with a body mass index (BMI) of 38 kg/m2. The patient was tachypneic on admission with a respiratory rate of 27/min and a spO2 of 88% at room air. Throat swab reverse transcriptase polymerase chain reaction (RT-PCR) for severe acute respiratory syndrome coronavirus 2 (SARS-CoV-2) was found to be positive. The patient was admitted to the COVID high dependency unit (HDU) and was initiated on supplemental oxygen therapy through a venturi mask (FiO2-40%). Serum ferritin and lactate dehydrogenase (LDH) were found to be within normal limits while D-dimer was elevated (2.21 g/L); C-reactive protein (CRP) was found to be 13.2 mg/dL -- a 10-fold elevation (Table 1). A chest X-ray was done and was found to have bilateral patchy opacities suggestive of viral pneumonia (Figure 1). The patient was initiated an injection methyl prednisolone 40 mg IV BD and an injection enoxaparin 80 mg subcutaneous (SC) twice a day which was continued for 10 days. The patient was diagnosed to have type 2 diabetes mellitus with glycated hemoglobin (HbA1c) of 7.2% and adequate glycemic control was achieved with an injection human insulin subcutaneous (SC). In addition, the patient also received an IV course of remdesivir for 10 days. During the course of their stay in the HDU, the patient improved symptomatically with no radiological worsening and hence was shifted to the ward. Supplemental oxygen therapy was tapered to 2 L/min delivered through nasal prongs. However, in the ward, the patient complained of acute onset right-sided pleuritic chest pain with worsening hypoxemia. Supplemental oxygen was given through a venturi mask (FiO2-60%). An urgent chest X-ray was done which showed a right-sided pneumothorax (orange arrow) with right lower lobe collapse (green arrow) (Figure 2). Subsequently, a CT thorax confirmed the presence of a massive right-sided hydropneumothorax (orange arrow) with features of a resolving / fibrotic stage (green arrow) of SARS-CoV-2 pneumonia (Figure 3). The patient was immediately shifted to the intensive care unit (ICU) and a 12 French intercostal drain (ICD) was placed (orange arrow) (Figure 4). Pleural fluid analysis revealed the presence of 68,300 white blood cells (WBCs) (85% polymorphs) and Gram stain of pleural fluid detected Gram-negative bacilli (Table 2). This confirmed a hospital-acquired superadded bacterial infection leading to hydropneumothorax. This patient likely suffered from a pneumothorax due to parenchymal damage secondary to SARS-CoV-2 infection and the prolonged course of steroids could possibly have precipitated a Gram-negative bacillus infection resulting in a hydropneumothorax. Oxygen was delivered through a high flow nasal cannula (FiO2-50%) and parenteral antibiotic therapy with injection piperacillin-tazobactam 4.5 g IV Q6th hourly was administered. The patient reported a marked improvement of symptoms following the insertion of ICD and initiation of appropriate antibiotic therapy. The ICD was removed after there was no air leak for more than 24 h. The patient was discharged after the completion of 10 days of parenteral antibiotic therapy. Chest radiography done at the time of discharge revealed adequate re-expansion of the right lung (Figure 5). The patient had a follow-up visit two weeks after discharge and reported no limitations in performing his daily activities.

 

 

**Table 1:** Investigations. BUN, blood urea nitrogen; CRP, C-reactive protein; LDH, lactate dehydrogenase

Lab parameter	Reference ranges for a 32-year-old man	SI unit	At admission
Hemoglobin	130-180 g/L	g/L	130g/L
Total count	3.50-11.00 x 10^9/L	10^9/L	3.1x10^9/L
Neutrophil:lymphocyte ratio			1.33(Poly:52.6%, Lymph:39.5%)
Platelets	150-450 x 10^9/L	10^9/L	150-450 x 10^9/L
Serum creatinine	59-104 µmol/L	µmol/L	70.72 µmol/L
BUN	2.9-8.2 mmol/L	mmol/L	2.86mmol/L
Na+	133-146 mmol/L	mmol/L	136mmol/L
K+	3.5-5.3 mmol/L	mmol/L	4.4mmol/L
Cl-	95-108 mmol/L	mmol/L	106mmol/L
HCO3-	22-29 mmol/L	mmol/L	24mmol/L
LDH	135-225 IU/L	IU/L	316 IU/L
Serum ferritin	30-400 µg/L	µg/L	60.7 µg/L
CRP	<5 mg/dL	mg/dL	13.2 mg/Dl
D dimer	<5 g/L	g/L	2.21 g/L
Procalcitonin	Procalcitonin <0.10 ug/L - antibiotics strongly discouraged Procalcitonin 0.10-<0.25 ug/L - antibiotics discouraged Procalcitonin 0.25-0.50 ug/L - antibiotics encouraged Procalcitonin >0.50 ug/L - antibiotics strongly encouraged	µg/L	0.40 µg/L

**Table 2:** Pleural fluid analysis. WBCs, white blood cells; LDH, lactate dehydrogenase; AFB, acid fast bacilli; MTB, Mycobacterium tuberculosis

Lab parameter	SI unit	Reference ranges	Values in our patient
Sugar	mmol/L	3.9–5.6 mmol/L	3.1 mmol/L
Protein	g/dl	1–2 g/dL	5 g/dL
Adenosine deaminase	IU/L	<43 IU/L	28.3 IU/L
Lactate dehydrogenase	U/L	<2/3^rd^ of the upper limit of normal serum LDH	707 U/L
WBCs		<1x10^9/L	68.3 x 10^9/L
Differential count			Polymorphs – 85% lymphocytes – 15%
Gram stain		Undetectable / negative	Gram negative bacilli
Culture and sensitivity		Undetectable / negative	No growth
AFB		Undetectable / negative	No AFB detected
Nucleic acid amplification test for Mycobacterium tuberculosis		Undetectable / negative	MTB not detected

## Discussion

Differential diagnosis

In a patient with COVID-19 infection and a prolonged HDU/ICU stay presenting with acute onset unilateral pleuritic chest pain, dyspnea, hypoxemia, and tachycardia the diagnosis of acute pulmonary thromboembolism should always be kept in mind. Pulmonary embolism has been reported to occur in COVID-19 patients causing an acute deterioration and it may even occur despite thromboprophylaxis [1]. In a recently published expert guidance on anticoagulation in COVID-19 patients with acute illness and low bleeding risk, it was suggested that a therapeutic dose of unfractionated or low molecular weight heparin might be more beneficial than standard dose anticoagulant prophylaxis [2].

Acute unilateral pleuritic chest pain with dyspnea and hypoxemia also points towards the possibility of a pneumothorax. A chest X-ray is crucial in such patients to rule out a pneumothorax. The occurrence of pneumothorax is rather uncommon in SARS-CoV-2 pneumonia. In a cohort of 3368 patients, only six of them (0.66%) developed pneumothorax [3]. Four out of the six patients were on mechanical ventilation [3]. The occurrence of pneumothorax in SARS-CoV-2 pneumonia in a patient with no positive pressure ventilation although rare should always be kept in mind. In a multicenter retrospective case series from the United Kingdom (UK), it was found that age >70 years and acidosis were associated with a poor prognosis [4]. In one study, which included SARS-CoV-2 patients with spontaneous pneumothorax, 13 out of 16 patients were non-smokers [5]. Our patient, however, had a 10-pack-year smoking history. The median time for the duration of development of pneumothorax in SARS-CoV-2 patients was 19 days [5]. A male preponderance of 88.8% has been observed in SARS-CoV-2 patients developing pneumothorax [6]. Though most reported cases of pneumothorax in SARS-CoV-2 patients occurred in those requiring oxygen and/or positive pressure ventilation, it may also occur in asymptomatic patients as reported by Rohailla et al. [7]. The pathologic changes in the lung parenchyma in SARS-CoV-2 pneumonia include cystic and fibrotic changes which may lead to alveolar breaks. This has been hypothesized to be the mechanism of pneumothorax in SARS-CoV-2 pneumonia. Prolonged coughing can also increase the intrathoracic pressure and is thought to contribute to the disease process to a certain extent [3]. A 61-year-old man in Germany, a non-smoker developed a right-sided tension pneumothorax 17 days after hospitalization. Some 20 days later, he developed a left-sided tension pneumothorax after a bout of coughing, without any evidence of a new infection. This supports the theory that elevated intrathoracic pressure from persistent and heavy coughing could contribute to the development of barotrauma complications in these patients [8]. The occurrence of hydropneumothorax has been described previously in SARS-CoV-2 pneumonia in only one patient and it usually indicates the occurrence of a secondary bacterial infection [9]. The treatment for SARS-CoV-2 pneumonia includes glucocorticoids for immunosuppression during the cytokine storm. However, clinicians should be cautious in its use and be vigilant towards the possibility of the occurrence of secondary infections in the susceptible patient. Our patient had a co-existing type 2 diabetes mellitus which could have increased his susceptibility to develop a secondary infection. However, it should be borne in mind that a patient on steroid therapy might not develop clinical features of a bacterial infection such as fever as it may be masked by steroid therapy [10]. Hence, clinicians should be judicious with the use of steroid therapy and be vigilant to detect possible secondary infections.

## Conclusions

In a COVID-19 patient with acute onset unilateral pleuritic chest pain, dyspnea, hypoxemia, and tachycardia the diagnosis of pneumothorax and pulmonary embolism should be kept in mind. The possibility of pneumothorax should be considered even in the resolving phase of COVID-19 infection. The absence of positive pressure ventilation does not preclude the occurrence of pneumothorax in a patient with COVID-19 pneumonia. The development of hydropneumothorax in a COVID-19 patient could be suggestive of a superadded bacterial infection. The treatment of SARS-CoV-2 pneumonia includes glucocorticoids and this may mask clinical features of an infection such as fever.
